# Takotsubo cardiomyopathy induced by pheochromocytoma: a case report

**DOI:** 10.1093/omcr/omad011

**Published:** 2023-02-27

**Authors:** Fabrice Boris Awadji, Bryan Richard Sasmita, Bi Huang, Yuying Han, Yuan Yang, Suxin Luo, Gang Liu

**Affiliations:** Department of Cardiology, the First Affiliated Hospital of Chongqing Medical University, Chongqing 400016, China; Department of Cardiology, the First Affiliated Hospital of Chongqing Medical University, Chongqing 400016, China; Department of Cardiology, the First Affiliated Hospital of Chongqing Medical University, Chongqing 400016, China; Department of Cardiology, the First Affiliated Hospital of Chongqing Medical University, Chongqing 400016, China; Department of Cardiology, the First Affiliated Hospital of Chongqing Medical University, Chongqing 400016, China; Department of Cardiology, the First Affiliated Hospital of Chongqing Medical University, Chongqing 400016, China; Department of Cardiology, the First Affiliated Hospital of Chongqing Medical University, Chongqing 400016, China

## Abstract

Pheochromocytoma presents various clinical manifestations and imprecise signs and symptoms. Along with other diseases, it is considered to be ‘the great mimic’. This is the case of a 61-year-old man who on arrival presented with extreme chest pain accompanied by palpitations, and with a blood pressure of 91/65 mmHg. An echocardiogram showed an ST-segment elevation in the anterior leads. The cardiac troponin was 1.62 ng/ml, 50 times the upper limit of normal. Bedside, echocardiography revealed global hypokinesia of the left ventricle, with an ejection fraction of 37%. Because ST-segment elevation myocardial infarction-complicated cardiogenic shock was suspected, an emergency coronary angiography was performed. It showed no significant coronary artery stenosis, while left ventriculography demonstrated left ventricular hypokinesia. Sixteen days after admission, the patient suddenly presented with palpitations, headache and hypertension. A contrast-enhanced abdominal CT showed a mass in the left adrenal area. Pheochromocytoma-induced takotsubo cardiomyopathy was suspected.

## INTRODUCTION

Pheochromocytoma crisis (PCC) is defined as a sudden increase of catecholamine, resulting in hemodynamic instability to the extent of multiple organ dysfunction syndrome [1, 2]. PCC is therefore a rare, life-threatening emergency. Due to its variable clinical manifestations, it may present as a cardiovascular complication, including ST-segment elevation myocardial infarction (STEMI), secondary takotsubo cardiomyopathy (TCM), transient hypotension, myocarditis, heart failure and hypertensive urgency [3, 4].

## CASE REPORT

A 61-year-old man was admitted following 5 days of fever and headache, coupled with 14 hours of aggravated chest pain. He had a medical history of hypertension and myocardial infarction, including a stent placement in the left anterior descending coronary artery at 54 years old. The patient was conscious upon arrival. His axillary temperature was 37.5°C, heart rate was 105 beats per minute (bpm), blood pressure was 91/65 mmHg and breathing was shallow at a rate of 40 breaths per minute.

**Figure 1:**
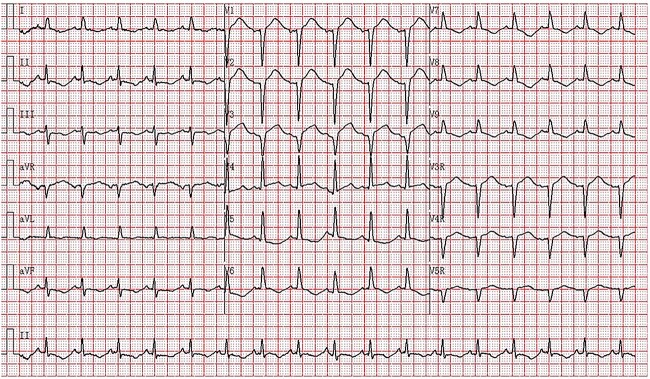
Fourteen hours after the onset of chest pain, the ECG showed sinus rhythm with a heart rate of 103 bpm, PR 127 ms, QRS complex of 72 ms, QTc 445mcs, axis −7°. Lead V1–V3 was (*r*) QS type. The ST segment in leads V1-V4 was elevated by 0.1–0.25 mV and depressed by 0.05–0.1 mV concurrently with T wave inversion in leads II, III, aVF and V5-V9.

Admission chest radiography and computed tomography (CT) pulmonary angiography demonstrated an enlarged heart with no evidence of infection or pulmonary embolism (Supplements 1 and 2). His echocardiography revealed global hypokinesia of the left ventricular wall with a reduced ejection fraction, left ventricular ejection fraction (LVEF) was 37%. Biomarker myocardial injury indicated an elevated cardiac troponin I at 1.62 ng/ml (normal range < 0.023 ng/ml). An electrocardiogram (ECG) showed an ST-segment elevation in the anterior leads (Fig. 1), which led us to suspect STEMI complicated by cardiogenic shock. Emergency coronary angiography (CAG) showed mild proximal right coronary artery stenosis, no left coronary artery stenosis and TIMI flow grade 2 (Supplements 3–5). Left ventriculography demonstrated left ventricular hypokinesia without the classic apical ballooning (Supplements 6 and 7). Subsequent biochemical tests revealed abnormal coagulation, elevated liver enzymes and kidney injury (Supplement 8).

**Figure 2:**
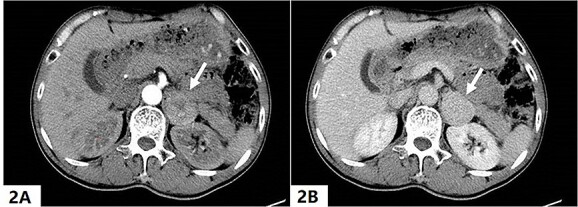
A quasi-round soft tissue mass with a size of about 3.7 cm (shown by arrow) was detected in the left adrenal region, the Hounsfield unit was about 76 in the arterial phase (2A) and 91 in the venous phase (2B).

**Figure 3:**
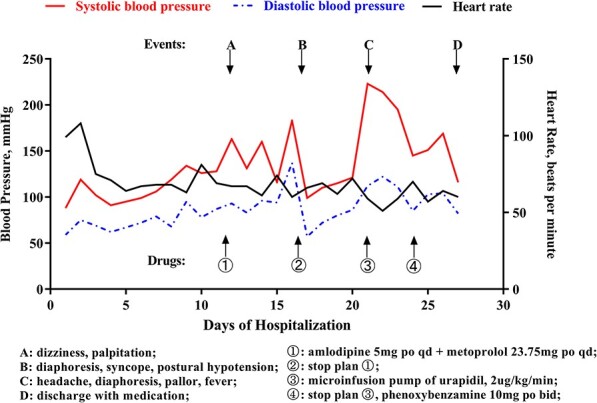
Main managements and events; changes in blood pressure, heart rate during hospitalization.

**Figure 4:**
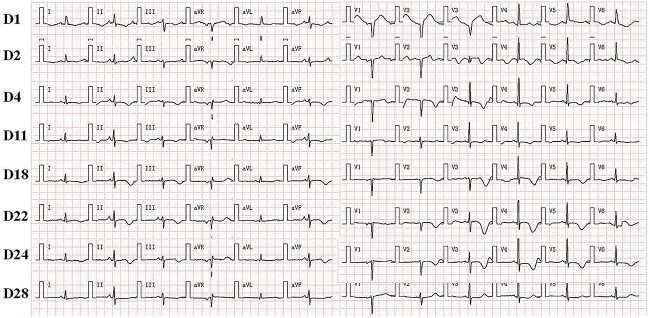
Dynamic ECG changes during hospitalization from Day 1 to Day 28. It shows the gradual recovery of ST-T segment starting from the administration of phenoxybenzamine.

**Figure 5:**
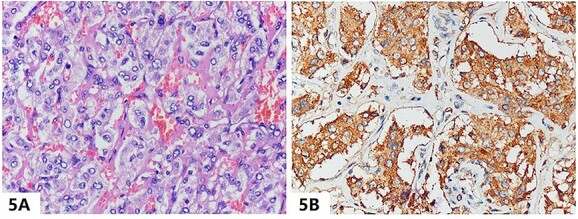
(A) The tumor resected from the left adrenal gland was presented as polygonal nests and eosinophilic cytoplasm on hematoxylin and eosin staining (magnification, ×400), and (B) immunohistochemical stains revealed a strong reaction to the neuroendocrine marker of chromogranin A (magnification, ×400), which are the typical features of pheochromocytoma.

On the 16th day after admission, the patient suddenly presented with dizziness, diaphoresis, palpitation, pallor and severe fluctuation in blood pressure. We suspected pheochromocytoma and performed a series of examinations. Plasma metanephrine was normal, while plasma normetanephrine was 2327.8 ng/L (normal range, 21.0–150.0 ng/L). Contrast-enhanced abdominal CT showed a mass (3.7 cm × 3.0 cm) in the left adrenal area (Fig. 2A and B). The patient was diagnosed with PCC and was given fluid resuscitation and an oral phenoxybenzamine (treatment strategies are presented in Fig. 3). Follow-up ECGs showed a gradual recovery of the ST-T segment (Fig. 4). The patient also received a laparoscopic resection under general anesthesia, and the histopathologic examination indicated pheochromocytoma (Fig. 5A and B). The post-surgery cardiac echocardiographic examination revealed a normal heart size and LVEF of 69%. At a 4-year follow-up, he had stable blood pressure and no recurrence of symptoms.

## DISCUSSION

PCC is a rare life-threatening condition with a high mortality (15–28%) [1]. The classic presentation of pheochromocytoma includes hypertension, palpitations and headache [2]. However, the variety of clinical presentations and imprecise signs and symptoms place PCC among other diseases that have been designated ‘the great mimic’ [5]. In this report, we present the case of an atypical variant of pheochromocytoma-induced takotsubo cardiomyopathy.

Pheochromocytoma-induced cardiovascular complication is rare and can easily be misdiagnosed. Although hypertension is the most common complication [6], pheochromocytoma has also been associated with transient hypotension, myocardial ischemia, ventricular tachycardia, takotsubo cardiomyopathy and heart failure [3, 4, 7]. Several mechanisms have been proposed to explain this phenomenon. They include a surge of endogenous catecholamine, increased myocardial oxygen demand, microvascular impairment and multivessel epicardial spasm [4, 7]. In our case, it is important to note that the patient presented with transient hypotension, which could be explained by impaired peripheral response to catecholamines, adrenocortical insufficiency, baroreflex failure and hypovolemia due to increased capillary permeability and diaphoresis [8]. Given these causal possibilities, clinicians should be aware of pheochromocytoma presenting with transient hypotension, which could indicate a severe underlying condition [9].

Catecholamine, particularly norepinephrine, is known to have a toxic effect on the myocardium. Norepinephrine released from the sympathetic nerve terminals can decrease myocyte viability through cAMP-mediated calcium overload, resulting in contraction band necrosis, a pathological hallmark of TCM [10]. The variation in the ratio of epinephrine to norepinephrine level has been associated with distortion in the conductive system of the heart [4], and with several ECG changes, such as deep symmetric T-wave inversion, prolongation of QT interval and ST-T segment abnormalities [4, 5]. Cheng *et al.* [5] demonstrated progressive QT prolongation and T-wave inversions which resolved after right adrenalectomy. These findings are similar to ours, in which we found that the T-wave inversions and QT interval gradually worsened during hospitalization, with a resolution of the ECG after administration of phenoxybenzamine and local resection of the adrenal tumor.

We initially suspected STEMI or TCM as the primary diagnosis. However, emergency CAG showed no significant coronary artery stenosis, while left ventriculography demonstrated left ventricular hypokinesia. Eventually, the classic triad of symptoms emerged, and enhanced abdominal CT revealed a mass in the left adrenal gland. Hence, we ruled out pheochromocytoma-induced myocardial injury as the primary cause. The following evidence and assumptions from our examination informed our clinical judgments. First was the unexplained transient hypotension in the absence of critical coronary artery stenosis. Second, elevated cardiac troponin and ST-segment elevation indicated the presence of myocardial injury. Third, progressive T-wave inversions and prolonged QT intervals could be attributed to the dynamic changes in catecholamine levels. Fourth, large regional wall motion abnormality is indicative of takotsubo syndrome. Fifth, post-operative cardiac echocardiography showed resolution of motion wall abnormalities, while follow-up ECGs noted the disappearance of QT prolongation and T-wave inversions. Based on these findings, we believe that pheochromocytoma-induced global TCM is the best explanation for our case.

## CONCLUSION

Left ventricular hypokinesia accompanied by transient hypotension or blood pressure fluctuation could signal the presence of a pheochromocytoma. Therefore, awareness of this rare condition is essential to avoid any delay in diagnosing such a serious but treatable disease.

## Supplementary Material

Supplement_1_omad011

Supplement_2_omad011

Supplement_3_omad011

Supplement_4_omad011

Supplement_5_omad011

Supplement_6_omad011

Supplement_7_omad011

Supplement_8_omad011

## Data Availability

The documents used during the current study are available from the corresponding author on reasonable request.
