# Supplementary figures and images for: Takotsubo cardiomyopathy induced by pheochromocytoma: a case report
Source: Oxf Med Case Reports. 2023 Feb 27;2023(2):omad011. doi: 10.1093/omcr/omad011 (PMC9969823; doi:10.1093/omcr/omad011)

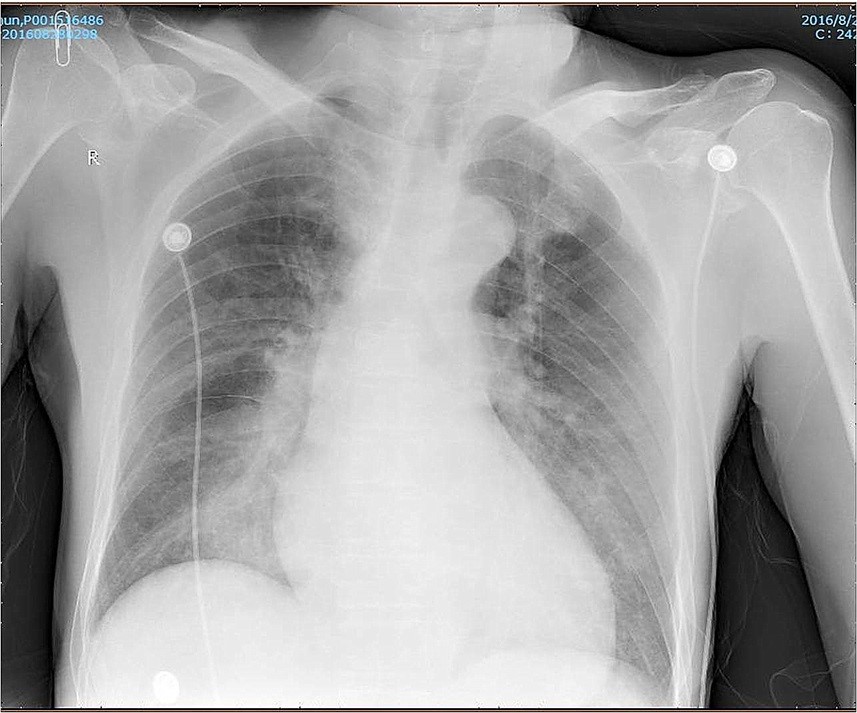

Supplement: Supplement_1_omad011 [file supplement_1_omad011.jpeg]

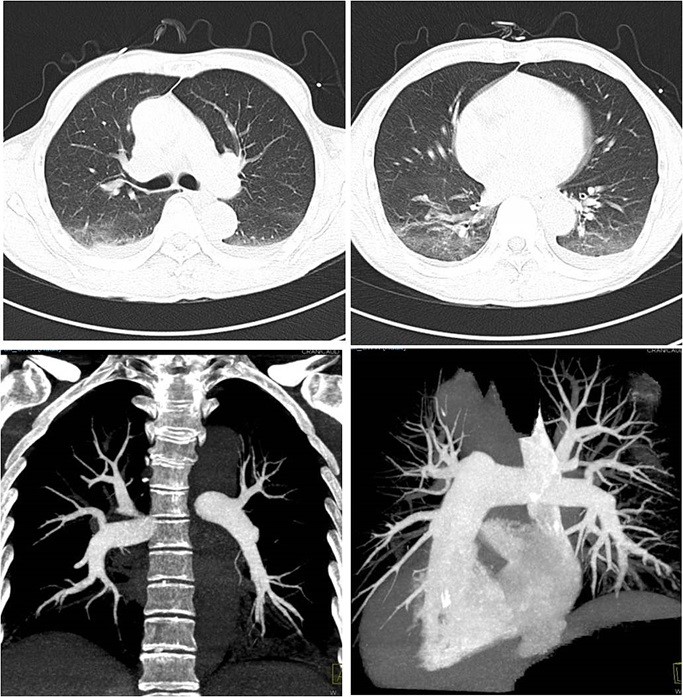

Supplement: Supplement_2_omad011 [file supplement_2_omad011.jpeg]
