# Supplementary material for: Takotsubo cardiomyopathy induced by pheochromocytoma: a case report
Source: Oxf Med Case Reports. 2023 Feb 27;2023(2):omad011. doi: 10.1093/omcr/omad011 (PMC9969823; doi:10.1093/omcr/omad011)
Supplement: Supplement_8_omad011 [file supplement_8_omad011.docx]

**Supplement 8. Admission Biochemical Tests**

| **Serum Levels** | **Patient Level** | **Normal Range** |
| --- | --- | --- |
| **WBC (*10^9^/L)** | **8.21** | **3.5—9.5** |
| **Hb (g/L)** | **146** | **130—175** |
| **PLT (*10^9^/L)** | **101** | **85—303** |
| **Hct (%)** | **43.3** | **40—50** |
| **ALT (U/L)** | **5735 ↑** | **9—50** |
| **AST (U/L)** | **＞7500 ↑** | **15—40** |
| **BUN (umol/L)** | **11.5 ↑** | **3.6—9.5** |
| **Cr  (umol/L)** | **255 ↑** | **57—111** |
| **Na+ (mmol/L)** | **136 ↓** | **137—147** |
| **K+ (mmol/L)** | **6.8 ↑** | **3.5—5.3** |
| **PT (s)** | **21.4 ↑** | **10.5—13.7** |
| **APTT (s)** | **41.1 ↑** | **15—34** |
